# Is There a Role for the Therapeutic Drug Monitoring of Colistin? An Overview

**DOI:** 10.3390/ph13030042

**Published:** 2020-03-06

**Authors:** Maria-Paula Avila, Tatiana Pacheco, Sara Arias, Rosa-Helena Bustos, Julio-Cesar Garcia, Diego Jaimes

**Affiliations:** Evidence-Based Therapeutics Group, Clinical Pharmacology, Universidad de La Sabana, 140013 Chía, Colombia; mariaaviga@unisabana.edu.co (M.-P.A.); tatianapacpa@unisabana.edu.co (T.P.); saraarvi@unisabana.edu.co (S.A.); julio.garcia@unisabana.edu.co (J.-C.G.); Diegojf@unisabana.edu.co (D.J.)

**Keywords:** Therapeutic Drug Monitoring (TDM), colistin, multiresistant bacteria

## Abstract

Colistin is used as a last-line antibiotic for the treatment of Gram-negative multiresistant bacteria. Due to its high nephrotoxicity, Therapeutic Drug Monitoring (TDM) is recommended for dose adjustment. We aimed to evaluate the available evidence of TDM in patients given colistin to treat Gram-negative infections. In this paper, we offer an overview, using an electronic search of the literature (published up to June 2019, without language restrictions) that compares the clinical outcomes and measurements of colistin TDM. Ultimately, the Therapeutic Drug Monitoring (TDM) of colistin in Plasma could prevent nephrotoxicity risk.

## 1. Introduction

Multidrug-resistant (MDR) infections have emerged as a treatment challenge [1]. MDR literally refers to resistance to more than one antimicrobial agent; however, different reviews to date have been ambiguous when specifying/concretizing this definition. Falagas et al. reviewed this classification’s variability in the pertinent literature, especially regarding *Pseudomonas aeruginosa* and *Acinetobacter baumannii*. Most define their resistance profiles as resistance to three or more classes of antibiotics [2]. Another categorization of this resistance phenomenon is illustrated by resistance to a key antimicrobial, such as *Staphylococcus aureus*, where resistance to methicillin represents a multi-resistance pattern [3]. Although new antibiotics are commercially available with in vitro activity for resistant Gram-positive organisms, such as vancomycin-resistant Enterococci and methicillin-resistant *S. aureus*, a limited armamentarium continues to exist for MDR Gram-negative infections, and the industry has been reluctant to invest in the research and development of these types of antibiotics [1]. 

**Figure 1:**
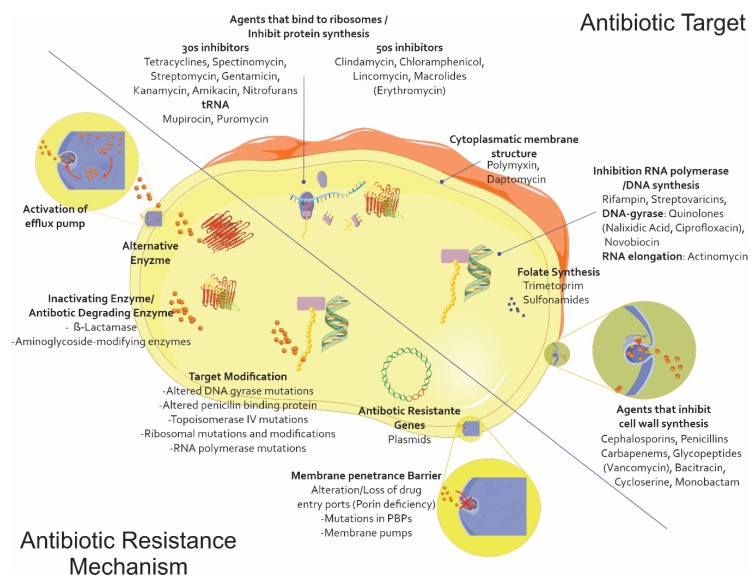
The main antibiotic resistance mechanisms. Alteration/modification of the target site of action with poor affinity for a particular antibiotic and the modifications being caused in the original PBPs. Impermeability and efflux (i.e., the appearance of mutants deficient in one or more outer membrane porins (OMP)) and nonspecific mutants affecting ß-lactams, quinolones, tetracyclines, and chloramphenicol. ß-lactamase production, i.e., the enzymatic hydrolysis of penicillins and cephalosporins, possibly reaching the carbapenems (the Ambler and Bush classifications). Vector graphics are licensed under the Creative Commons Attribution 3.0 Unported License and Service Medical Art [5,6].

Due to the lack of a new therapeutic arsenal compared to what is available for treating infections caused by Gram-positive germs, also including the spectrum for which the polymyxin family has regained a relevant role, the extensive family of β-lactam antibiotics is the main antibiotic family used for treating infections caused by this type of microorganism; resistance to them represents the major limitation in choosing an appropriate treatment. This phenomenon has partly arisen due to the excessive use of this type of antibiotic on humans and animals, enabling strong selection pressure through its subsequent elimination in the environment and leading to the evolution and grouping of multiple resistance mechanisms [4] (Figure 1). 

**Figure 2:**
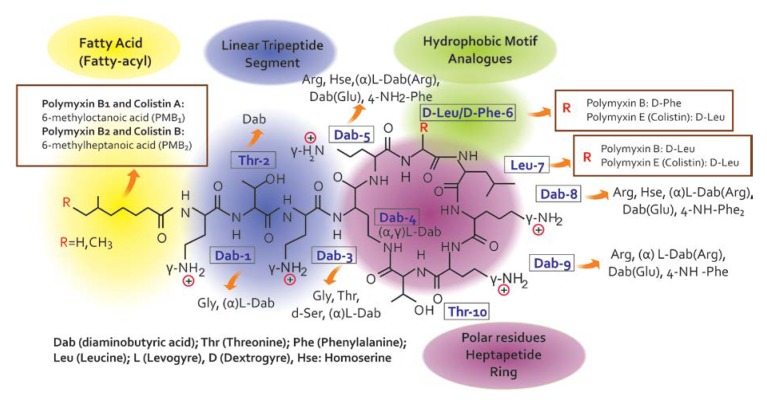
Chemical structure of colistin and polymyxin B [22].

Colistin is a venerable drug that fell into disuse in 1970. However, polymyxins have been reconsidered in clinical practice to treat patients with infections caused by multidrug-resistant Gram-negative bacterial [7] infections [8,9]. Since there are no promising chemical entities for these infections, the medical community has been forced to reappraise the clinical value of colistin, considering its use in many cases, especially in intensive care settings, as a salvage therapy [10]. Colistin belongs to the family of Polymyxins that were first isolated in 1947 from the soil bacterium, *Paenibacillus polymyxa* subsp. *Colistinus* [11]. Five chemically different compounds have been designed and recognized (polymyxin A, B, C, D, and E) [12]. Due to their important association with nephrotoxicity, only polymyxin B (PMB) and E (PME, i.e., colistin) are clinically available [11,13]. Colistin is only commercially in the form of its inactive prodrug, colistin methanesulfonate (CMS), which must be hydrolyzed in vivo to active colistin that is conducive to their significant pharmacokinetic differences in vivo [14,15]. Colistin’s chemical structure difference (Figure 2) between PMB and PME occurs in position 6, which is occupied by the D-Phe in PMB and D-Leu in colistin [14,16].

There are two commercial formulations of colistin: colistin sulfate and colistimethate sodium. The latter is a prodrug that hydrolyzes to colistin and is used intravenously. It has a wide distribution, except at the synovial, pleural, and pericardial levels, with a volume specific to healthy (1.24 L/kg) and critically ill (0.72 L/kg) individuals. It presents moderate protein binding in 50% of patients, with the hydrolysis metabolism of colistimethate to the active form of colistin found in <30%, with a half-life of elimination of 2–3 h through glomerular filtration.

According to the Institute for Clinical and Laboratory Standards (CLSI), susceptibility to *P. aeruginosa* and *A. baumannii* is defined by a minimum inhibitory concentration (MIC) <2 mg / L [17]. This suggests that the plasma concentration of colistin is between 1 to 5 µg/mL [18]. However, although the dose regimen is not clear for the populations of some patients, such as patients with different renal statuses, it is important to investigate more of the pharmacokinetic properties of this patient population [19]. The lack of pharmacological information related to the administration of colistin in a critical state prevents the administration of an optimal dose regimen that reconciles adequate antibacterial activity with minimum toxicity. It is expected that the pharmacokinetics of colistin will be dramatically altered in critical patients because they are frequently prone to large oscillations in the volume of their distribution, fluctuations in renal clearance, and variable protein binding. Likewise, the antibacterial activity of colistin is attenuated in high bacterial loads, such as pneumonia [20]. Polymyxin E is administered intravenously in the form of a CMS prodrug and converted to plasma colistin, which finally exerts a therapeutic effect at >72 h and has been used as an inclusion criterion in studies evaluating the dose of colistin. The results of these studies offer reasonable nonempirical indications of postantibiogram therapy [21].

Pachecho et al. have described the mechanisms of action of colistin resistance related to LPS modifications, repulsion mechanism, remodeling membrane, and modifications to OM porins, and overexpression of efflux pump systems [22]. The WHO mentions that these mechanisms are defined within acquired resistance to colistin [23]. The increase in the colistin resistance rat has been found in both humans and the environment around us. This resistance is mainly given by Gram-negative bacteria that differ with the strains, with K. pneumoniae and *A. baumannii* showing high resistance rates. For the first case, *Klebsiella pneumoniae* has shown an increase of 3.57% to 9.68% from 2002 to 2013, respectively, in a study developed in Tunisian [24]. Other studies report a high resistance rate of 43% in Italy, although this percentage differs in other regions such as China or Dubai [25,26,27]. On the other hand, for *A. baumannii*, the MARRON study (multicenter epidemiological surveillance study of the antibiotic resistance of nosocomial pathogens) the CoR in Russia was 1.9% [28], similar to the EARS-Net 2013 study, where the average resistance rate was 5% from Greece and Italy [29]. However, the percentage of resistance varies in different populations, where the SENTRY study (2006–2009) showed a resistance percentage of 30.6% in Korea. [30].

The adequate usage of colistin in a clinical setting remains an enigma, especially because colistin was never subjected to the modern drug development and regulatory approval processes [31]. This fact led to an overall lack of reliable modern data regarding its pharmacokinetic/pharmacodynamics (PK/PD) and adequate translational information between in vitro findings and clinical outcomes [22,32]. To overcome the risk of the spread of plasmid-mediated resistance to colistin that could compromise the last antibiotic bastion [33], an international consensus has emerged [34]. 

The most recent study published in January 2019 provides 35 recommendations for the optimal use of polymyxins [35]. Special attention is given to the role of Therapeutic Drug Monitoring (TDM). TDM enables the quantification of the toxicity, changes of PK, and determination of the narrow therapeutic index (NTI) in drugs. We hypothesized that, given its narrow therapeutic window, the measurement of the plasma colistin concentration to guide therapy may be beneficial [34]. The objective of this paper was to evaluate the available evidence on the necessity for TDM in patients treated with colistin to treat Gram-negative infections.

## 2. Data Sources and Search

Published articles and conference abstracts (published until 31 January 2019) that reported the clinical outcomes of monitoring colistin serum concentrations were identified through computerized literature searches in PubMed, EMBASE, Scopus, and the Web of Sciences and the Cochrane Library. The references of the retrieved articles were also searched for additional studies. The search terms applied to the algorithm included a combination of text free terms and Medical Subject Headings (MeSH) as follows: (“colistin” MeSH) OR (“Colistin”[Mesh]) AND “colistinmethanesulfonic acid” (supplementary concept) AND (“therapeutic drug monitoring” OR “TDM” OR “drug monitoring” OR “therapeutic monitoring” OR “serum concentration monitoring” OR “therapeutic drug” OR “drug monitoring” MeSH). No restriction on language was applied.

## 3. Study Selection Criteria

Two reviewers independently searched the literature and examined the relevant studies for further assessment of the data. Each reviewer was blinded to the other reviewer during the process of data extraction. Both randomized controlled trials (RCTs) and observational studies comparing clinical outcomes of TDM in patients treated with colistin were eligible. The following types of studies were excluded from the analysis: reviews, editorials, guidelines, and studies focusing only on pharmacokinetic and pharmacodynamic abstracts in animals or pediatric patients.

## 4. Data Extraction and Outcomes

The data extracted from the identified studies included the author, year of study and publication, country in which the study was conducted, study design, number of patients enrolled, population characteristics (type and etiology of infection), clinical efficacy, microbiological efficacy, defined as successful eradication of the causative pathogens, overall mortality, nephrotoxicity, duration of colistin therapy, and length of hospital stay. The primary outcome of this paper was clinical efficacy. This was generally defined in the individual studies as the absence of symptoms and signs by infections. Secondary outcomes included the duration of colistin therapy, length of stay, 30 days mortality, and nephrotoxicity. Nephrotoxicity was defined by the RIFLE (acronym of Risk, Injury, and Failure; and Loss; and End-stage kidney disease) Criteria during colistin therapy. 

## 5. Discussion

**Figure 3:**
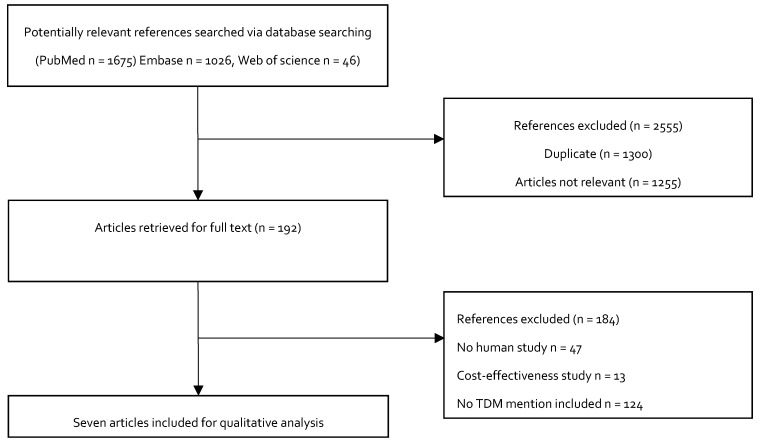
Flowchart depicting the selection process for the studies included in the paper.

Figure 3 shows the study selection process for inclusion in the study. We initially identified 2747 potentially relevant studies. A total of 2555 articles were excluded after a review of the titles: 1300 were duplicate articles, and 1255 were not relevant, because they did not describe the doses of colistin as loading doses or maintenance doses during treatment. The full-text articles of the remaining 192 studies were evaluated. Another 184 studies were excluded. Among these, 122 of the studies did not mention TDM, 13 studies were cost effectiveness studies, and 49 were animal studies. Seven studies were ultimately included in the qualitative analysis. 

To study the characteristic data, the patients (aged between 18 and 88 years) received intravenous colistin for their infections by multidrug resistance Gram-negative bacilli. The variables described included the pathogen and susceptibility of the isolate as multidrug-resistant (MDR) or extensively drug-resistant (XDR), as well as the loading and daily dose in million units, the therapeutic drug monitoring peak and trough levels, and nephrotoxicity as an adverse drug reaction. 

There are two commercial formulations of colistin: colistin sulfate and colistimethate sodium. The latter is a prodrug that hydrolyzes to colistin and is used intravenously. It has a wide distribution, except at the synovial, pleural, and pericardial levels, with a volume that is specified according to whether the individuals are healthy (1.24 L/kg) or critically ill (0.72 L/kg). It has moderate protein binding in 50% of patients, with the hydrolysis metabolism of colistimethate yielding the active form of colistin in <30%, with a half-life of elimination of 2–3 h through glomerular filtration.

There is a problem regarding its dosage, due to the variety of administration units according to certain geographical areas, such as Europe, the United Kingdom, and India, where colistimethate is expressed in international units (IU). However, in America, Asia, and Australia, colistin is expressed in milligrams. Thus, the equivalence of 1 MUI corresponds to 30 mg of colistin, and this equals approximately 80 mg of colistimethate. Thus, a given dose of colistin will correspond to 2.7 times the number of milligrams of colistimethate. 

The most common infection warranting the use of colistin is pneumonia, followed by a urinary tract infection, bloodstream infection, surgical site infection, and other infections less frequent, as shown in Table 1. The most commonly included pathogen was *P. aeruginosa* in five of the studies, followed by *A. baumannii*. The equivalent concentration of colistin is 0.4 mg of CBA (Colistin Base Activity) for 1 mg of CMS and 12.5 million IU of CMS. 

Further, in the treatment with colistin, most studies reported their doses in million units (MU), and three studies reported colistin in milligrams >2.5 mg/kg/day, which is equivalent to 75,000 U/kg/day for colistin’s base activity, and three studies reported using a loading dose >6 MU for each patient. A summary description of the included studies is reported in Table 1. All studies reported on patients treated between 2013 and 2019. We did not find any randomized control trials; four studies were cohort studies, and four were case reports. The most commonly included pathogen was *P. aeruginosa* in five of the studies, followed by *A. baumannii*.

In general, studies detailed that there are similarities in the areas of the infectious process and the types of Gram-negative bacteria present. It is important to note that, in most of the research on the use of colistin, the presentation of nephrotoxicity prevails in the first six days of treatment in the patients who are administered a loading dose. This leads the authors included in this review to hypothesize the existence of other factors due to the absence of the measurement of therapeutic drug monitoring (TDM) in other studies, which could be affecting the pattern of routine use of loading doses according to the recommendations established. 

Regarding the limitations of this type of review [43], it should be borne in mind that there are few available studies on the monitoring of plasma parameters for this antibiotic and its relationship with a favorable clinical outcome in patients with multidrug-resistant bacteria. Higher doses have a high risk of presenting nephrotoxicity, as well as higher mortality and therapeutic failure in greater proportion, which could be considered a new field of research for this medicine. The therapeutic levels can be measured for the risk management and prevention of documented nephrotoxicity, with plasma concentrations of colistin >2.5–3 mg/L [7,18,19,20]. Hence, this nephrotoxicity is reversible, with improvement shown when discontinuing treatment or decreasing the established dose.

## 6. Conclusions

In this study, the variability of the maintenance dosage of colistin was considered. There has been a violation of its representation, with the results of its plasma factors outside the expected therapeutic range. Therefore, the studies described characterize the heterogeneity of these results, mainly due to the different methodologies used. It is advisable that, in future lines of research, a greater number of patients with those missing data be included in the variables of interest, in order to estimate more representative samples of the population using colistin. 

In conclusion, the results of this paper indicate that monitoring colistin plasma data could prevent the nephrotoxicity described in drug-safety studies for therapeutics. 

## Figures and Tables

**Table 1:** The main characteristics of the studies included.

Ref	Design, Year, Country	Type of Infection	Pathogen and Susceptibility of the Isolate (MDR–XDR)	n	Loading Dose (MU)	Dose (MU/day)	Peak Level (µg/mL)	Trough Level (mg/mL)	Clinical Efficacy	ADR: Nephrotoxicity
[36]	Case Report 2013 Germany	Borderline Syndrome	*Pseudomonas aeruginosa MDR*	1	Yes 9 MU	Days 1–6: 9 MU	0.91	0.48	Inadequate	Non
						Days 6–9: 18 MU	1.1	0.5	Adequate	↓ eGFR
						Days 9–21: 9 MU	7.1	4.2	Adequate	↓ eGFR CVVHDF
						Days 21–28: 6 MU	0.97	0.5	Inadequate	Multi-organ failure
[37]	Prospective cohort 2011–2015 Spain	Bronchial infections (21), UTI (11) Pneumonia (7) Skin infections (6) Bacteremia (10)	*Pseudomonas aeruginosa MDR* 59 cases (92.2%)	64	Yes 6 MU	6	NI	>2.42 (n = 7) <2.42 (n = 57)	NI	>2.42 (mg/mL) Nephrotoxicity more frequent and earlier
[38]	Prospective observational 2013 Spain *	Pneumonia 24 Acute bronchitis 23 Urinary tract infection 15 Skin and soft tissue infection and surgical site infection 15 Bacteremia 5 Others 20	*Pseudomonas aeruginosa* MDR:89 *Acinetobacter baumannii* MDR:9 *Klebsiella pneumoniae* MDR: 1	102	NI	3 (n = 28)	0.65 (0.24–1.99)	0.71 (0.2–2.01)	n = 25 adequate	AKI day 7 = 11 AKI EOT = 21
						6 (n = 42)	1.13 (0.15–5)	1.14 (0.11–5)	n = 32 adequate	AKI day 7 = 11 AKI EOT = 21
						9 (n = 16)	1.84 (0.5–6.62)	1.84 (0.45–5.99)	n = 11 adequate	AKI day 7 = 7 AKI EOT = 9
						Others (n = 16)	1.5 (0.16–3.7)	1.5 (0.16–3.7)	n = 11 adequate	AKI day 7 = 6 AKI EOT = 11
[39]	Case Report 2017 Germany	Intracranial infection	*Acinetobacter Baumannii* MDR	1	YES 10 MU	9MU IV Plus intraventricular 0.5 MU	4.4	1.36	Adequate	AKI day: 7
[40]	Case Report 2015 Japan	Bacteremia	*Pseudomonas aeruginosa MDR*	1		2.5 mg/kg	NI	Day 13 = 7.88	Adequate and microbiological efficacy (>1.36 mg/L)	Day 13: eGFR = 23.8
[41]	Prospective Cohort 2009–2013 Spain **	Pneumonia and ITU	*Pseudomonas aeruginosa* *XDR*	91	NI	3–9 mg/kg	NI	1.49 ± 1.4	Adequate n = 72	AKI day 7: n = 18 AKI EOT: 33
								2.42 ± 1.49	Inadequate n = 19	AKI day 7: n = 12 AKI EOT: 16
[42]	Prospective Cohort 2019 Korea **	Pneumonia and ITU	*Acinetobacter Baumannii* XDR	15	NI	5 mg/kg	5.50 ± 2.75	2.29 ± 1.15	Adequate	AKI day 7: n = 4

* Median (interquartile range). ** Mean + DS. **NI:** not indicated; **MIU:** million units; **µg/mL:** micrograms/milliliters; **mg/kg:** milligrams/kilograms; **MDR:** multidrug resistant; **XDR:** extensively drug-resistant; **ADR:** adverse drug reactions; **(eGFR):** estimated glomerular filtration rate; **(CVVHDF):** continuous venovenous hemodiafiltration; **AKI:** Acute Kidney Injury; **EOT:** end of treatment; **IV:** intravenous. **Adequate:** absence of symptoms and signs by infection. **Inadequate**: did not answer to treatment with colistin because of persistent symptoms or clinical failure. **↓**: decrease.
